# A Further Test of the Impact of Online Gaming on Psychological Wellbeing and the Role of Play Motivations and Problematic Use

**DOI:** 10.1007/s11126-019-09656-x

**Published:** 2019-08-05

**Authors:** C. Goh, C. Jones, A. Copello

**Affiliations:** 1 School of Psychology, University of Birmingham, Edgbaston, B15 2TT UK; 2 Birmingham and Solihull Mental Health Foundation Trust, Edgbaston, UK

**Keywords:** Multiplayer Online Battle Arena (MOBA), Online games, Problematic gaming, Motivators for playing, Escapism, Self-esteem, Self-efficacy

## Abstract

The impact of increased online gaming play time on psychological wellbeing was examined focusing on the Multiplayer Online Battle Arena (MOBA) game genre. This relationship was explored with respect to motivators for playing and resilience factors. A cross-sectional, online questionnaire design was employed with participants (*N* = 165) to examine the relationship between weekly average hours played and psychological wellbeing. Five previously reported motivators for playing were tested as mediating variables. In addition, exploratory analyses were conducted to determine the moderating effects of self-esteem and self-efficacy on ‘escapist’ gaming and psychological wellbeing. Results revealed a significant correlation with higher levels of play time associated with poorer psychological wellbeing. This relationship was partially mediated by ‘escapist’ motivation. Self-esteem was found to moderate the negative impact of ‘escapist’ gaming on psychological wellbeing. Research and the associated clinical implications are discussed.

## Introduction

The number of people using online platforms for gaming has increased exponentially during the last decade; a report by ComScore [6] suggested that almost 217 million people play games online annually. With the proliferation of online games, there has been increasing concern over the excessive use of gaming and the ramifications on psychological wellbeing. The most extensively researched online gaming genre in the recent literature involves Massively Multiplayer Online Role Playing Games (MMORPGs). Studies have demonstrated that low self-esteem, depressive symptoms, problematic gaming behaviour, and poorer general health result from the problematic use of MMORPGs (e.g. [35, 38, 46]). Kuss et al. [25] suggested that the immersive properties found in MMORPG gameplay, namely escapism (i.e. avoidance of real-life problems), role-playing (i.e. adopting an in-game persona), and character customisation (i.e. personalisation of character), are associated with more problematic outcomes. At present it is unclear whether the reported impact is specific to MMORPG’s or a more general effect of problematic use of computer gaming.

The Multiplayer Online Battle Arena (MOBA) gaming genre has experienced a rapid rise in popularity over the past five years [37]. The shift from MMORPGs to MOBA games is evident through MOBA’s greater active player population and international recognition as a competitive sport. Even though MOBA games share features with MMORPGs (such as multiplayer online interaction and single character control), MOBA games are distinct in terms of their game mechanisms and lack the role-playing element found in MMORPGs, with the MMORPG having a strong investment in character development and encouraging immersion during long periods of in game-play. In contrast, MOBA involves relatively short team matches (30-60 min) after which the players characters are reset.

It is not known if playing MOBA games, which are distinct from MMORPGs in terms of its game mechanics, are likewise associated with poorer psychological outcomes. It is therefore important to explore the association between playing online games and psychological wellbeing in the context of other gaming genres such as MOBAs, and whether other factors influence this relationship.

### Impact of Online Games

Research in online MMORPG gaming tends to emphasise its negative impact on psychological wellbeing, with studies suggesting that MMORPG players are more vulnerable to negative psychological and physical outcomes [25, 35, 28, 30, 34]. Less attention has been given to the positive impact of online gaming on psychological wellbeing, such as helping players develop social skills, foster a social support network, enhance positive affect, and improve wellbeing [10, 19, 29, 51, 53]. It is imperative to further evaluate the relationship between online gaming and psychological wellbeing, as there may be important factors that can influence the direction and strength of this relationship.

### Motivations for Playing

Demetrovics et al. [9] posit that behaviour is largely determined and influenced by motives. The investigation into motivational factors is not new and has been examined in the field of addiction studies. For example, motivations for drinking were found to account for 50% of the variance in adolescent alcohol use [26]. Accordingly, understanding the motives and needs underlying why individuals play online games may lead to greater insight into the relationship between excessive use and psychological wellbeing.

Kahn et al. [23] developed a scale examining players’ motivations for playing games in the context of MOBA and MMORPG gaming genres. The authors argued that while other scales exist (e.g. [54]); they are limited in terms of their focus on specific gaming genres or lack of behavioural validation. Kahn et al. [23] conducted an exploratory factor analysis for items taken from past scales assessing the motivations for playing various video games [45, 54]. The analysis revealed six factors reflecting six distinct types of players: ‘socialisers’ (i.e. playing to socialise with others); ‘completionists’ (i.e. playing to complete every aspect of the game); ‘competitors’ (i.e. playing to win); ‘escapists’ (i.e. playing to escape from real life); ‘story driven’ (i.e. playing because of the story development); and ‘smarty-pants’ (i.e. playing to enhance their intelligence). The scale was thereafter validated on both the MOBA and MMORPG gaming genres.

‘Escapist’ players are defined as individuals who use gaming to escape from real life [23, 27]. Several studies highlighted that the motivation for escapism and immersive gaming are associated with negative psychological and social outcomes (e.g. [4, 24, 48]). Griffiths [20] identified that adults who engaged in gaming to escape from real-life problems experienced negative consequences on wellbeing compared to adults who played to socialise. Similarly, Stetina et al. [48] discovered that while ‘escapist’ gaming may act as a coping strategy for dealing with real-life difficulties, this was associated with more problematic outcomes. More recently, Kirby et al. [24] identified the role of players’ motivation as mediating the relationship between the amount of time spent playing MMORPGs and psychological wellbeing. Yee’s [54] three motivators for playing MMORPGs (Achievement, Social Interaction, and Immersion) were tested as mediators between play time and psychological wellbeing. The study found that increased play is associated with poorer psychological wellbeing, specifically where there is greater player motivation for immersion and escapism, *which points towards a negative association between using games to escape from real-life problems and poor mental health.*

Nonetheless, the negative outcomes associated with ‘escapist’ gaming may be mitigated by resilience factors. Literature on resilience factors has demonstrated how self-esteem can act as a defence mechanism by protecting individuals from experiences that are harmful [36, 47, 50]. Thoits [50] postulates that self-esteem protects individuals from threats to the self while attempts to resolve the problem are made. Similarly, self-efficacy, which is having the self-belief that one can overcome challenging environmental demands, has been demonstrated to promote effective coping strategies when dealing with stressors in life [1, 22]. Conversely, individuals with low self-esteem and/or self-efficacy may be more prone to the negative effects of ‘escapist’ gaming. Accordingly, it would be important to examine the potential of such resilience factors in moderating the impact of ‘escapist’ gaming.

## Study Aims

The aim of the present study was therefore to examine the relationship between the amount of time spent playing DOTA 2 (a popular MOBA) and psychological wellbeing. Five^1^ out of six Kahn et al. [23] motivations for play were tested as mediating variables. Overall, it was hypothesised that:

1. Greater time spent playing DOTA 2 would be associated with poorer psychological wellbeing.
2. In line with the findings from Kirby et al. [24], Khan et al.’s (2015) ‘escapist’ motivator for playing would mediate the association between play time and psychological wellbeing.
3. Using Khan et al. (2015) motivators for playing, the mediating effect of ‘socialisers’, ‘completionists’, ‘competitors’, and ‘smarty-pants’ will be examined.

Additionally, the potential moderating effects of self-esteem and self-efficacy between motivation for play and psychological wellbeing was explored.

## Method

### Participants

Participants were people who perceived themselves to be regular DOTA 2 players and were recruited by online advertisements on gaming websites. There was no restriction placed upon play time across the sample of players. The inclusion criteria were:

1. Adults, aged eighteen and over, who perceive themselves as regular DOTA 2 players;
2. Fluency in the English language, in order to be able to accurately and appropriately fill in the questionnaires;
3. A score of less than seven on the short version of the Social Desirability Scale.

One hundred and sixty-five participants^2^ took part in the study (155 males and ten females). To control for response bias, participants who scored seven or above on the Social Desirability Scale were excluded from further analyses (*n* = 16, 9.7%). Analyses were conducted for 149 participants (142 males and 7 females).

### Measures

Play time was assessed via self-report, in terms of gameplay days per week, hours per day and hours per week. The distribution for play time was positively skewed with the median play time at 18 h per week.

#### Psychological Wellbeing

Goldberg and Hillier’s [15] General Health Questionnaire (GHQ-28) was used as measure of Psychological Wellbeing. The questionnaire contains four subscales; Somatic Problems, Anxiety and Insomnia, Social Functioning and Depression. Each item is scored on a four-point Likert scale (from zero = no difficulties to three = much greater difficulties than usual). A higher score is indicative of poorer psychological wellbeing. The Cronbach’s Alpha Score for the scale is 0.9 [11].

#### Motivations for Play

Kahn et al.’s [23] Trojan Player Typology scale is a 15-item questionnaire used to identify players’ motivation for playing DOTA 2. Play motivators include ‘socialisers’, ‘completionists’, ‘competitors’, ‘escapists’, ‘story driven’, and ‘smarty-pants’. Participants were required to record their extent of agreement with statements relating to various aspects of gameplay. Each item is scored on a five-point Likert scale, where zero implied ‘strongly disagree’ and five implied ‘strongly agree’. As mentioned, the ‘story-driven’ motivation was removed following pilot testing due to its lack of relevance to DOTA 2 players. Khan et al. (2015) reported that the Cronbach’s Alpha reliability coefficients for each motivation are ‘Socialisers’ (.69), ‘Completionists’ (.67), ‘Competitors’ (.75), ‘Escapists’ (.70), and ‘Smarty-pants’ (.79) respectively.

#### Self-Esteem

Rosenberg’s [42] Self-Esteem Scale was used as a measure of self-esteem. The scale is a ten-item questionnaire in which respondents indicate on a four-point Likert scale (from one = strongly disagree to four = strongly agree) the extent of their agreement of the statements. Higher scores indicate higher self-esteem. Rosenberg [42] reported that the scale had good internal consistency (.77). A varied selection of independent studies using such samples as– parents, men over 60, high school students, and civil servants, − showed alpha coefficients ranging from 0.72 to 0.87.

#### Self-Efficacy

Schwarzer and Jerusalem’s [44] Generalised Self-Efficacy Scale was used. The self-efficacy scale is a ten-item questionnaire, in which respondents indicate on a four-point Likert scale (from one = not true at all to four = exactly true) the extent of agreement with the statements. A higher score is indicative of higher self-efficacy. The scale has good internal consistency (.82 to .93) for the samples studied.

#### Social Desirability

The short version of the Social Desirability Scale (SDS; [49]) was used. The scale includes ten true/false statements designed to reveal social desirability in the respondent. Higher scores indicate a greater tendency to present oneself in a positive manner. Fischer and Fick [12] reported that the short version of the SDS has good internal consistency (.88) and is highly correlated with the original scale (.96) developed by Crowne and Marlowe [7].

### Procedure

The research study was advertised to players of DOTA 2, both via game forums (internet webpages set up for groups of players to discuss issues) and social media sites (e.g. DOTA 2 Facebook pages). The survey was undertaken online using the Limesurvey survey programme [43]. Demographic questions were presented first, followed by the General Health Questionnaire, the Motivations for Play Questionnaire, the Rosenberg Self-Esteem Scale, the Generalised Self-Efficacy Scale, and the Social Desirability Scale.

### Data Analysis

The distribution for play time was positively skewed with the median play time at 18 h per week. Accordingly, nonparametric statistical procedures have been used were possible. Bootstrap confidence intervals are robust to the violation of parametric assumptions [33]. Therefore, bootstrap CIs were used for inferential tests. Unless otherwise stated, bootstrap results are based on 5000 bootstrap samples and the bias corrected and accelerated bootstrap confidence intervals are provided.

A zero-order correlation was used to determine the relationship between the independent variable (average number of hours spent playing per week) and the dependent variable (psychological wellbeing). To determine the potential mediating effect of motivations for playing the Preacher and Hayes’ [39, 40] model of mediation was used. In addition to calculating multiple mediator values simultaneously, the model handles the violation of parametric inference assumptions by reporting bias corrected and accelerated bootstrap confidence intervals. Preacher and Hayes [39, 40] have argued that this method is more robust than non-parametric statistical procedures and has a higher statistical power than the standard Sobel Test of mediation.

## Results

### Sample Characteristics

The participants’ demographic information is presented in Table 1. Participants originated from 30 countries although the largest number was from the UK (28%).

**Table 1:** Summary of sample demographic variables

	Categories	N (%)
Gender	Male	142 (95.3)
	Female	7 (4.7)
Relationship Status	Single	114 (76.5)
	Married	5 (3.4)
	Cohabiting	21(14.1)
	Divorced	1 (0.7)
	Other	8 (5.4)
Employment	Full time employment	44 (30)
	Part time employment	19 (12.8)
	Unemployed	8 (5.4)
	Student	77 (51.7)
	Stay at home parent	1 (0.7)

The pattern of DOTA 2 play for the sample is summarised in Table 2. The majority of participants indicated that they had been playing the game for more than four years (34.2%). Eighty-four percent of participants reported having played the game for two years or more. The mean level of game experience recorded by participants was level 100.28 (SD = 56.9), which indicates that they had invested a substantial amount of time in playing DOTA 2.

**Table 2:** Pattern of play

		Mean (SD)	Range
Age		23.2 (4.67)	18–44
Play Pattern	Hours per week	22.74 (16.19)	1–70
	Years playing		<1- > 4
	Days per week	5.22 (1.69)	1–7
	Hours per day	3.99 (2.26)	1–10
Game experience	Experience level	100.28 (56.89)	0–435

The total score of the GHQ represents overall wellbeing and a higher score is indicative of poorer psychological wellbeing. The mean GHQ score for the sample was 21.80 (SD = 12.90) and scores ranged from 3 to 56. The presence of mental health difficulties was determined according to the procedure described by Goldberg et al. [16]. Using this method, the Likert scores (0–1–2-3) were recoded (0–0–1-1) and a total score exceeding the cut-off of six was considered indicative of ‘caseness’. In the sample, 27.5% (*n* = 41) of the participants exceeded the cut-off for caseness.

No significant correlations were found between age and the total GHQ score (r = −0.051, 95% CI [−0.19 to 0.09]), the Somatic Problems subscale (r = −0.02, 95% CI [−0.15 to 0.12]), the Anxiety and Insomnia subscale (r = −0.05, 95% CI [−0.20 to 0.11])), the Social Functioning subscale (r = −0.04, 95% CI [−0.17 to 0.10]), and the Depression subscale (r = −0.06, 95% CI [−0.19 to 0.09]).

Rosenberg’s Self-Esteem Scale [42] was used to measure global self-worth. A high score is indicative of higher self-esteem. The mean score was 27.97 (SD = 5.10) and scores ranged from 17 to 40. There was no significant correlation found between self-esteem and age (r = 0.06, 95% CI [−0.10 to 0.20]).

The General Self-Efficacy Scale (1995) identifies individuals’ belief in terms of their ability to respond to difficult situations or setbacks. A higher score represents higher self-efficacy. A mean score of 29.75 (SD = 5.46) was obtained and the scores ranged from sixteen to 40. Age was not found to be significantly correlated with self-efficacy (r = 0.15, 95% CI [−0.06 to 0.31]) (Table 3).

### The Association between Play Time and Psychological Wellbeing

The impact of play time on psychological wellbeing was evaluated in terms of the relationship between the average number of hours played per week and the total GHQ score. A significant positive correlation was obtained between play time and GHQ scores (r = 0.43, 95% CI [0.28 to 0.57]). Results suggest that as the amount of play time per week increased, the greater the GHQ scores were (i.e. poorer psychological wellbeing), accounting for approximately 18% of the variance.

Significant positive correlations were also found for each of the GHQ subscale scores; Somatic Problems (r = 0.40, 95% CI [0.22 to 0.55]), Anxiety and Insomnia (r = 0.36, 95% CI [0.20 to 0.50]), Social Functioning (r = 0.32, 95% CI [0.17 to 0.47]), and Depression (r = 0.37, 95% CI [0.22 to 0.52]). Altogether, the results indicated that a greater number of hours spent playing DOTA 2 is associated with poorer levels of overall psychological wellbeing.

### Mediation Analysis

Five mediated paths were included in the Preacher and Hayes’ [39, 40] mediation model (‘socialisers’, ‘completionists’, ‘competitors’, ‘escapists’, and ‘smarty-pants’). The significance of the mediated pathways was evaluated via bootstrap bias corrected and accelerated confidence intervals of the path coefficients of the model.

In the unmediated null model, the effect of play time on psychological wellbeing was β = 0.35, 95% CI [0.23 to 0.46]. This was reduced to β = 0.22, 95% CI [0.12 to 0.32] when the mediating effect of the motivators were included. Therefore, the sum of the indirect effects within the mediation model was β = 0.12, 95% CI [0.03 to 0.22].

**Fig. 1:**
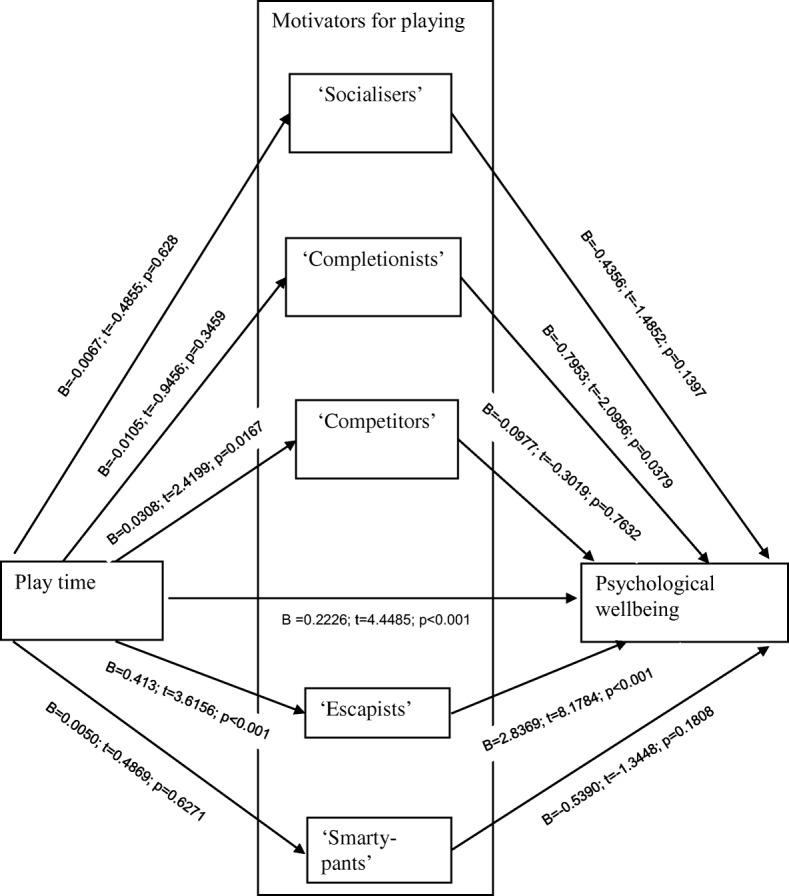
Mediation model with statistical effects of pathways

Figure 1 illustrates the individual path coefficients for each motivator for playing and the associated significance tests. In terms of the overall mediation effects, the ‘socialiser’ motivator was not found to be significant (β = 0.003, 95% CI [−0.01 to 0.03]). Similarly, the paths mediated by the ‘completionist’ motivator (β = 0.01, 95% CI [−0.01 to 0.04]), ‘competitor’ motivator (β = −0.003, 95% CI [−0.03 to 0.02]), and ‘smart-pants’ motivator (β = −0.003, 95% CI [−0.02 to 0.01]) did not show a significant overall effect. Consistent with the findings of Kirby et al. [24], only the path mediated by the ‘escapist’ motivation yielded a significant overall effect (β = 0.12, 95% CI [0.05 to 0.20]). Accordingly, a partial mediation effect was observed (Fig. 1).

**Fig. 2:**
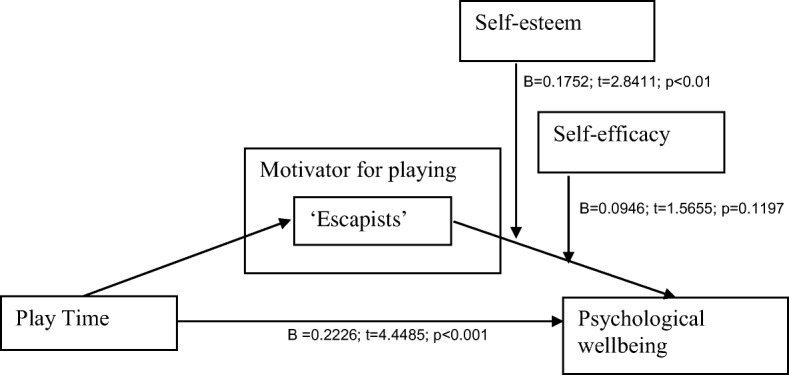
Moderator model with statistical effects of pathways

These results indicated that the ‘escapist’ motivation significantly mediated the relationship between play time and psychological wellbeing. The positive beta value indicates that the greater number of hours spent playing, alongside increased escapism, is related to poorer psychological wellbeing. However, the association between play time and psychological wellbeing was not fully mediated by the ‘escapist’ motivator, indicating that there may be other variables not accounted for by the model that mediate this relationship.

An exploratory analysis was thereafter conducted to determine whether resilience factors such as self-esteem and self-efficacy moderated the negative impact of the ‘escapist’ motivator for playing on psychological wellbeing (Fig. 2). Preacher and Hayes’ [39, 40] mediator moderator analysis procedure was used to explore the potential interaction effect between self-esteem (and self-efficacy) and escapism on psychological wellbeing. In order to aid the interpretation of the interaction effect, the self-esteem was reverse coded so that a positive score would indicate low levels of self-esteem. The product of self-esteem and the ‘escapist’ motivation was then calculated. The same procedure was replicated for the construct of self-efficacy and its interaction with escapism.

The results yielded a positive interaction effect between escapism and self-esteem (β = 0.18, 95% CI [0.05 to 0.30]). It can therefore be concluded that the relationship between the ‘escapist’ motivator for play and psychological wellbeing is moderated by the level of self-esteem. The positive beta value indicates that individuals with low self-esteem who play the game to escape have poorer psychological wellbeing overall.

The mediator moderator analysis however did not reveal a significant interaction between escapism and self-efficacy (β = 0.09, 95% CI [−0.02 to 0.21]). The results suggest that the relationship between the ‘escapist’ motivator for play and psychological wellbeing is not moderated by self-efficacy.

## Discussion

The present study aimed to examine the relationship between time spent playing Multiplayer Online Battle Arena (MOBA) games and psychological wellbeing, with motivators for playing as potential mediating factors. The study’s results suggest that increasing play time is associated with poorer psychological wellbeing, and that this relationship is mediated by the ‘escapist’ motivation. Further, the study found that low levels of self-esteem, alongside increasing escapism, were associated with poorer psychological wellbeing.

From the sample, 27.5% of participants met criteria for a diagnosable mental health problem. This is a slightly elevated statistic compared to the prevalence rates of mental health difficulties in the UK population, estimated to be around 17% of the population [32]. The higher proportion of online gamers suffering from mental health difficulties is likewise reflected in other studies examining mental health amongst online gamers (e.g. [24, 52]).

A significant positive correlation was found between the hours spent playing DOTA 2 per week and GHQ scores; a higher number of hours spent playing the game was found to be associated with poorer psychological wellbeing. A moderate effect size and 18% of the variance in psychological wellbeing can be explained by the amount of time spent playing the game. Similarly, the association between GHQ subscale scores and psychological wellbeing explained 10% to 16% of the variance. The results suggest that there is an increased likelihood of mental health difficulties amongst individuals who invest a significant amount of time playing DOTA 2. While the moderate effect size provides some evidence for mental health risks associated with increased play time, it cannot fully explain why some individuals who invest as much time into the game do not experience negative outcomes.

Kahn et al. [23] motivators for playing were considered in the context of this relationship. Specifically, five motivators were tested as mediators; ‘socialisers’, ‘completionists’, ‘competitors’, ‘escapists’ and ‘smart-pants’. Previous research has found that individuals who play MMORPGs as a means to escape from real-life difficulties are at most risk of negative outcomes [3, 4, 20, 24, 28]. The primary aim of the present study was to see if this association could be replicated in the MOBA gaming genre. The findings from the present study demonstrated that playing DOTA 2 as a means for escapism mediated the relationship between play time and psychological wellbeing. The remaining four motivators for play did not mediate the relationship between play time and psychological wellbeing.

The results from the present study and from Kirby et al.’s [24] study suggest that ‘escapist’ gaming may be used as an avoidant coping strategy for real-life difficulties [48]. The link between avoidant coping strategies and negative outcomes has been studied in the literature; avoidant coping strategies have been shown to have an association with negative consequences, such as depression or increased stress [2, 21]. Similarly, the literature on problem gambling has established a link between escapism and negative outcomes [8, 41]. Accordingly, when an individual uses gaming as a coping mechanism to escape from real-life problems, the association between longer play time and poorer mental health is strengthened.

**Table 3:** Summary of test variables

		Mean (SD)
GHQ-28	Total	21.80 (12.9)
	Somatic	4.85 (3.26)
	Anxiety & Insomnia	5.44 (4.11)
	Social functioning	7.26 (3.06)
	Depression	4.23 (4.93)
Self-Esteem	Total	27.97 (5.10)
Self-Efficacy	Total	29.75 (5.46)
Play Motivation	Socialisers	10.56 (2.7)
	Completionists	11.75 (2.19)
	Competitors	10.48 (2.55)
	Escapist	6.02 (2.34)
	Smarty-pants	6.17 (2.01)

Unlike the expansive virtual worlds in MMORPGs and the long-term investment in a single character, MOBA games only offer a single virtual arena and character statistics reset after a victory or a loss. The research in MMORPGs appears to suggest that the escapist properties are more pronounced given the opportunity for immersion in a virtual realm and the experience of a different life through a game character [54]. However, the current study’s findings challenge this conclusion; these findings suggest that playing MOBA games (that do not offer the kind of expansive virtual environment or character immersion akin to that of MMORPGs), is still associated with poorer mental health when an individual plays the game to escape from real life. The present study identified that the relationship between play time and psychological wellbeing is not fully mediated by the ‘escapist’ motivation. This suggests that there are other variables contributing to this relationship that are not accounted for by the model. Kirby et al.’s study [24] reported that there was no direct effect of play time on psychological wellbeing when the mediated variables were accounted for (i.e. character customisation, escapism and problematic use). There may be other variables not accounted for in the model used in the present study that likewise mediate the relationship between play time and psychological wellbeing, which could be explored in future research.

In terms of the role of resilience factors, self-esteem was found to be a moderator for escapism and mental health. In other words, the negative effects of using games to escape from real-life problems are influenced by an individual’s self-esteem. Martyn-Nemeth et al. [31] reported that low self-esteem was associated with avoidance coping and depressive mood. Similarly, Chapman and Mullis [5] reported that adolescents with lower self-esteem utilised more avoidant coping strategies compared to adolescents with higher self-esteem. It is possible that individuals who already suffer from low self-esteem play online games as a way to avoid real-life threats to themselves, and this form of coping, via avoidance, leads to poorer mental health. There may however be a host of alternative explanations regarding the way self-esteem influences the relationship between escapism and psychological wellbeing. Self-efficacy was not found to moderate the relationship between escapism and psychological wellbeing.

While the present study is similar to other studies focusing on the MMORPG genre in terms of a sample comprising of predominantly male online gamers (e.g. [17, 24, 54]), the gender distribution was extremely skewed towards males in the present study (20:1). The majority of the sample was from the UK and it is not known whether results would be generalisable to online gamers in different geographic locations, as motivations for play may vary based on cultural factors. The present study excluded participants under the age of eighteen years. However, the impact of games on age may vary for children and adolescents (e.g. [18]). While a correlational design was selected for the present study, a longitudinal study design is more appropriate for conclusions on causality to be drawn. A previous study that employed a longitudinal design found that the existence of greater impulsivity, in addition to a larger amount of time spent playing, together with low social competence, were risk factors for pathological gaming, contributing in turn to poorer mental health outcomes [14].

### Summary of Conclusions

The present study aimed to shed some light on the relationship between play time in MOBA games and psychological wellbeing. While some evidence exists showing that increased play time is associated with poorer psychological wellbeing, the ‘escapist’ motivator for playing was found to mediate this relationship. Increased MOBA gameplay was associated with poorer psychological wellbeing, specifically where there is greater motivation for escapism. The association between ‘escapist’ gaming and poor psychological wellbeing was strengthened for individuals with low self-esteem.

## Footnotes

1 The ‘story-driven’ sub-scale did not appear to be relevant to DOTA 2 players as the game is not story driven.

2 Based on the calculations of Fritz and MacKinnon [13], a sample size of 71 participants allows for the identification of medium sized mediation effects (i.e. with path coefficients of at least .39), with a power of 0.80.
